# Basic biochemical characterization of cytosolic enzymes in thymidine nucleotide synthesis in adult rat tissues: implications for tissue specific mitochondrial DNA depletion and deoxynucleoside-based therapy for TK2-deficiency

**DOI:** 10.1186/s12860-020-00272-3

**Published:** 2020-04-28

**Authors:** Liya Wang, Ren Sun, Staffan Eriksson

**Affiliations:** Department of Anatomy, Physiology and Biochemistry, Swedish University of Agricultural Sciences, SE-750 07 Uppsala, Sweden

**Keywords:** Thymidine kinase 2, Thymidylate synthase, p53R2, Mitochondrial DNA depletion, dTMP synthesis; mtDNA, Mitochondrial DNA; RNR, Ribonucleotide reductase

## Abstract

**Background:**

Deficiency in thymidine kinase 2 (TK2) or p53 inducible ribonucleotide reductase small subunit (p53R2) is associated with tissue specific mitochondrial DNA (mtDNA) depletion. To understand the mechanisms of the tissue specific mtDNA depletion we systematically studied key enzymes in dTMP synthesis in mitochondrial and cytosolic extracts prepared from adult rat tissues.

**Results:**

In addition to mitochondrial TK2 a cytosolic isoform of TK2 was characterized, which showed similar substrate specificity to the mitochondrial TK2. Total TK activity was highest in spleen and lowest in skeletal muscle. Thymidylate synthase (TS) was detected in cytosols and its activity was high in spleen but low in other tissues. TS protein levels were high in heart, brain and skeletal muscle, which deviated from TS activity levels. The p53R2 proteins were at similar levels in all tissues except liver where it was ~ 6-fold lower. Our results strongly indicate that mitochondria in most tissues are capable of producing enough dTTP for mtDNA replication via mitochondrial TK2, but skeletal muscle mitochondria do not and are most likely dependent on both the salvage and de novo synthesis pathways.

**Conclusion:**

These results provide important information concerning mechanisms for the tissue dependent variation of dTTP synthesis and explained why deficiency in TK2 or p53R2 leads to skeletal muscle dysfunctions. Furthermore, the presence of a putative cytosolic TK2-like enzyme may provide basic knowledge for the understanding of deoxynucleoside-based therapy for mitochondrial disorders.

## Background

**Fig. 1:**
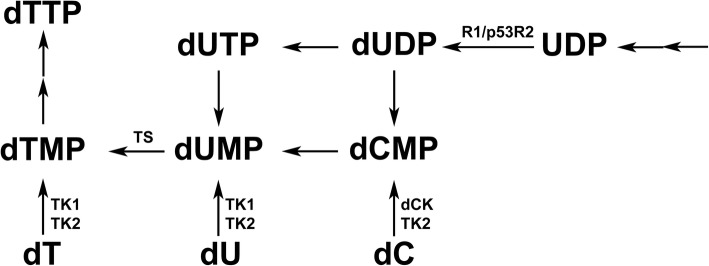
Schematic presentation of dTMP synthesis pathways. TK1, cytosolic thymidine kinase 1; TK2, thymidine kinase 2; TS, thymidylate synthase; dCK, deoxycytidine kinase; R1, ribonucleotide reductase large subunit; p53R2, p53 inducible ribonucleotide reductase small subunit

Thymidylate (dTMP) is an essential building block of DNA and synthesized by the salvage and the de novo pathways (Fig. 1). In the salvage pathway dTMP is produced by thymidine (dT) phosphorylation catalysed by thymidine kinases (TK1 and TK2) and in the de novo pathway by deoxyuridylate (dUMP) methylation catalysed by thymidylate synthase (TS). TK1 is a cytosolic enzyme expressed mainly in proliferating tissues [1, 2]. Mitochondrial TK2, on the other hand, is constitutively expressed [3, 4]. Determination of TK1 and TK2 activity in tissue or cell extracts is complicated due to the fact that both enzymes have overlapping substrate specificity [5–7]. Cytosolic deoxycytidine kinase (dCK), mainly expressed in lymphoid tissues, catalyzes the phosphorylation of deoxycytidine (dC) to dCMP, which can be further phosphorylated to dCTP or deaminated to dUMP and used for dTMP synthesis (Fig. 1) [8, 9]. In post mitotic tissues ribonucleotide reductase (RNR) activity is minimal since the expression of the small subunit is S-phase specific [10, 11]. However, the presence of a p53 inducible RNR small subunit (p53R2) enables the de novo pathway to provide dNTPs even for non-cycling cells [12]. Furthermore, TS activity is a prerequisite for the de novo synthesis of dTMP. In rodents both TK1 and TS activity are developmentally down-regulated and decreased to minimal levels within 2 weeks after birth [13, 14]. To our knowledge the levels of TS and p53R2 and the distribution of cytosolic deoxynucleoside kinases in adult animal tissues have not been reported.

Deficiencies in TK2 or p53R2 cause fatal myopathy and/or encephalomyopathy in humans [15, 16]. Tissue specific mtDNA depletion has also been observed in TK2 H126N knock-in and TK2 knock-out mice as well as p53R2 knock-out mice [16–18]. Defects in nucleotide metabolism have also been linked to nuclear genome instability and premature aging [19–21].

To understand why TK2 and p53R2 deficiency led to mtDNA depletion syndromes (MDS) we studied the levels and distribution of enzymes in dTMP synthesis in major adult rat tissues. A cytosolic isoform of TK2 was partially purified and characterized in addition to the mitochondrial TK2. TS activity and the levels of TS protein, as well as the levels of p53R2 protein were determined.

## Results

### Isolation of mitochondrial and cytosolic fractions

Mitochondrial and cytosolic fractions were prepared from fresh adult rats tissues according to standard procedures [22]. The quality was accessed by western blot analysis using antibodies against cytochrome c oxidase subunit 4 (COX4), which is located in the mitochondrial inner membrane; and cytochrome c, which is loosely associated with mitochondrial inner membrane. Both COX4 and cytochrome c were detected in all the mitochondrial preparations but also in the cytosol preparation of heart tissue (Fig. S1A). The absence of COX4 and cytochrome c in the cytosol preparations of brain, liver, kidney, spleen, lung and skeletal muscle demonstrated minimal contamination by mitochondria. The presence of COX4 and cytochrome c in the heart cytosol preparation indicated the presence of mitochondria, most likely due to that the isolation procedure was unable to release interfibrillar mitochondria from heart tissue as described earlier [23]. Therefore, ultracentrifugation was used to remove all mitochondria from heart cytosol preparation and in the final heart cytosol preparation no cytochrome c was detected (Fig. S1B).

However, the lack of cytochrome c and COX 4 in the cytosol preparations cannot exclude leakage of mitochondrial matrix proteins into the cytosol during preparation. Therefore, the mitochondrial matrix protein citrate synthase activity was assayed in both the mitochondrial and cytosolic extracts. As shown in Table 1 citrate synthase activity was mainly detected in mitochondrial fractions and the citrate synthase activity detected in cytosolic fractions was approximately 5–9% of that in the corresponding mitochondrial fractions. These results strongly indicated that there was minimal leakage of mitochondrial protein into the cytosolic fractions.

**Table 1:** Citrate synthase activity in mitochondrial and cytosolic preparations

Citrate synthase activity (μmol/min/mg)			
Tissue	Mitochondrial	Cytosolic	Cytosolic/mitochondrial
Liver	12.1 ± 0.5	0.70 ± 0.04	0.057
Heart	54.3 ± 1.3	5.02 ± 0.18	0.092
Brain	45.8 ± 3.4	3.13 ± 0.06	0.068
Kidney	28.3 ± 1.2	2.23 ± 0.09	0.078
Spleen	41.8 ± 1.0	2.03 ± 0.03	0.048
Lung	61.0 ± 1.7	3.50 ± 0.06	0.057
Skeletal muscle	31.0 ± 1.2	2.6 ± 0.18	0.083

### Identification and characterization of a cytosolic TK2-like enzyme

**Fig. 2:**
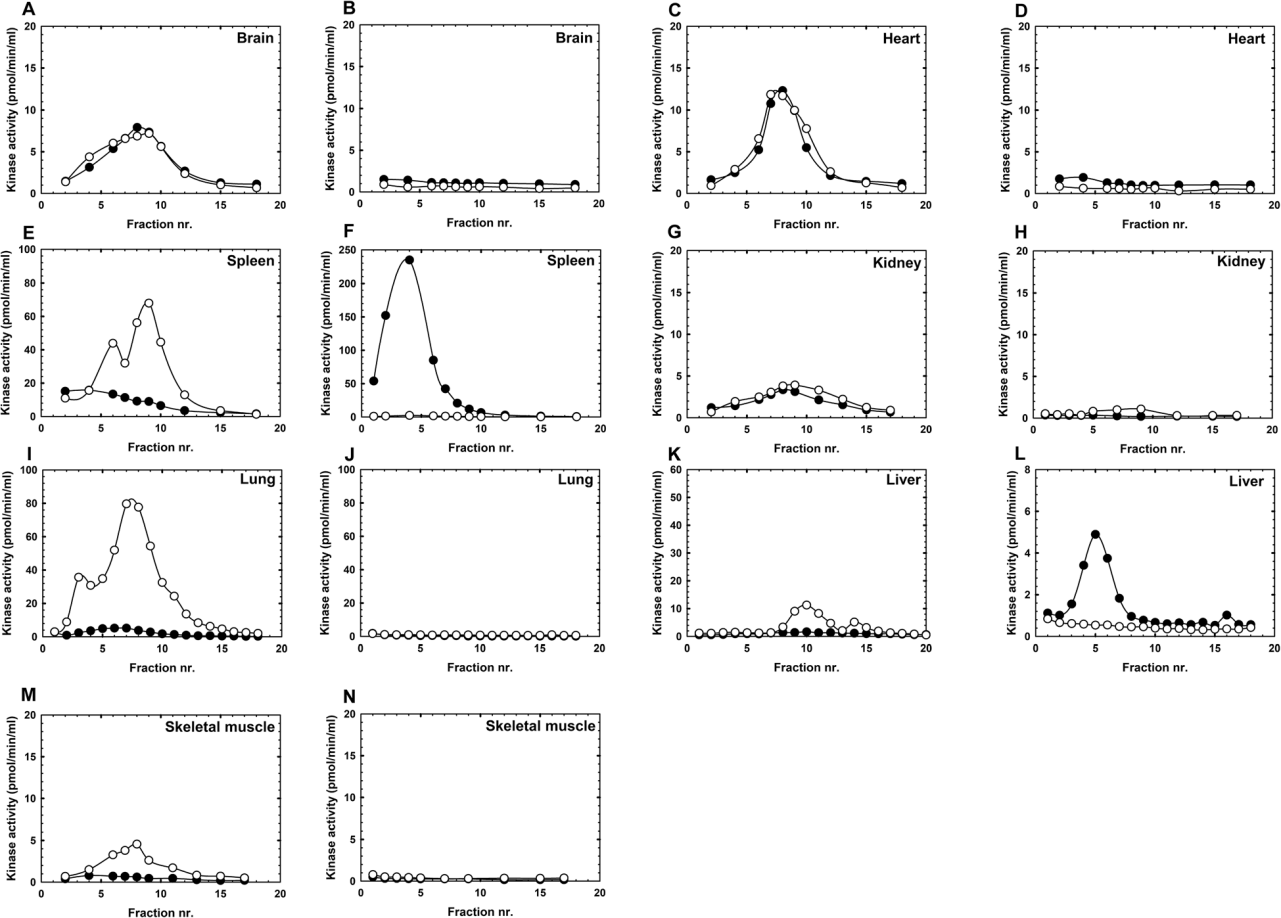
Distribution of cytosolic deoxynucleoside kinases in adult rat tissues determined by anionic (**a**, **c**, **e**, **g**, **i**, **k** and **m**) and cationic (**b**, **d**, **f**, **h**, **j**, **l**, and **n**) exchange column chromatography. Cytosolic proteins from each tissue were applied to a anionic exchange (DEAE-sephadex) column and the flow through (unbound protein) was applied to a cationic exchange (CM-sepharose) column. The bound proteins were then eluted with KCl gradient. Fractions were collected and assayed using tritium labeled dT (filled circles) and dC (open circles) as substrate. TK2 and dCK bind to DEAE-Sephadex column and TK2 eluted at low KCl concentration and dCK eluted at higher KCl concentration. TK1 binds to CM-Sepharose column and eluted at low KCl concentration

TK2 is known as mitochondrial enzyme. Since both dT and dC phosphorylating activities were present in the cytosolic fractions from all tissues (Table S1), we wanted to clarify which enzymes are responsible for the observed dT and dC phosphorylating activities. The cytosolic proteins were separated by anionic (DEAE-Sephadex) and cationic (CM-Sepharose) exchange column chromatography. The bound proteins were eluted with a linear KCl gradient. In brain, heart, spleen, kidney, lung, and skeletal muscle both dT and dC phosphorylating activity co-eluted in the same DEAE chromatography fractions (Fig. 2a, c, e, g, i and m), at concentration range similar to the elution profile of mitochondrial TK2 [24]. Under these conditions cytosolic dCK, if present, eluted with higher KCl concentrations [25], as shown in DEAE chromatograms from spleen, lung, liver, and skeletal muscle (Fig. 2e, i, k and m). TK1 protein, however, does not bind to the DEAE-Sephadex column but binds to the CM-Sepharose column and eluted at low KCl concentration [26], as shown in the liver and spleen chromatograms (Fig. 2f and l).

**Fig. 3:**
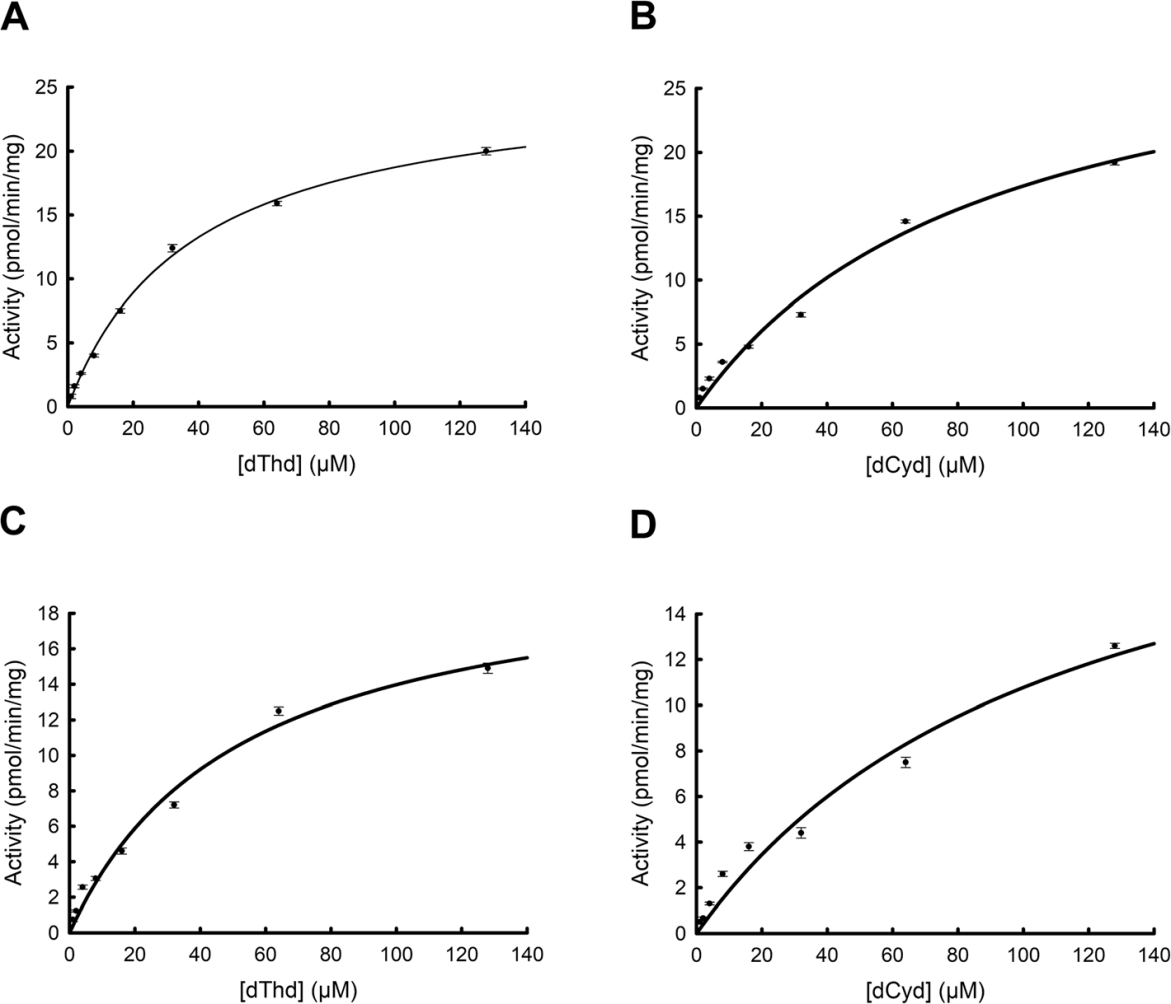
Substrate saturation curves of partially purified cytosolic TK2-like enzyme. Substrates used were [^3^H]-dT and [^3^H]-dC. Heart (**a** and **b**) and brain (**c** and **d**)

The DEAE fractions containing the dT and dC phosphorylating activity from heart and brain were pooled and characterized further. The heart enzyme had apparent K_M_ values for dT and dC of 38 ± 1.9 and 88.9 ± 12 μM and V_max_ values of 25.9 ± 0.5 and 32.8 ± 2.4 nmol/min/mg, respectively (Fig. 3a and b). The brain enzyme had apparent K_M_ values of 52.5 ± 6.1 (dT) and 113.1 ± 23 μM (dC) and V_max_ values of 21.3 ± 1.1 (dT) and 23.1 ± 2.8 (dC) nmol/min/mg, respectively (Fig. 3c and d). Thus, the heart and brain cytosolic TK2-like enzymes showed similar substrate specificity to the native and recombinant mitochondrial TK2 [26–28].

These results demonstrated the presence of a putative cytosolic TK2-like enzyme in all tissues except liver, and the presence of dCK in spleen, lung, liver, and skeletal muscle, and TK1 in liver, spleen, brain and heart. To our knowledge this is the first time it is shown in the cytosols from major tissues in adult animals.

### Comparison of mitochondrial and cytosolic thymidine kinase activities

**Fig. 4:**
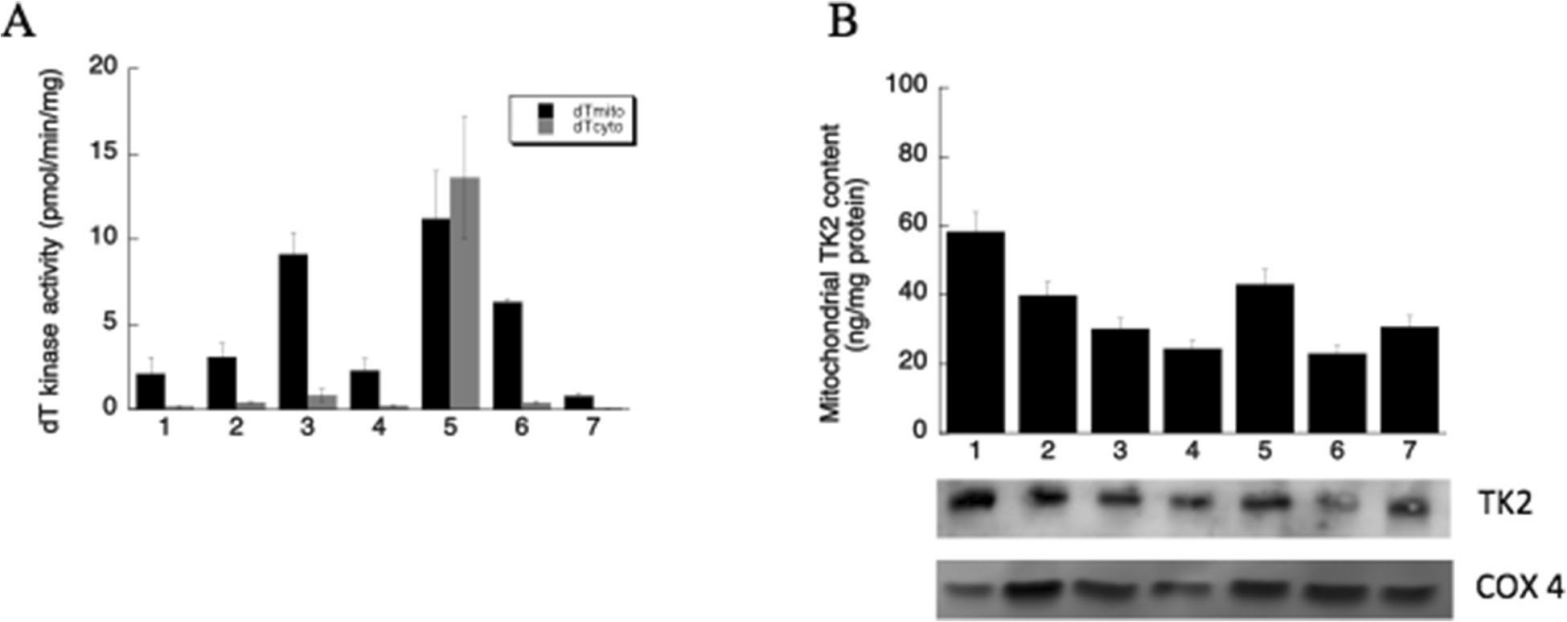
Mitochondrial and cytosolic thymidine kinase activities in rat tissues (**a**) and Mitochondrial TK2 protein levels (**b**). Mitochondrial TK2 protein levels were determined by immunoprecipitation followed by western blot analysis using TK2 specific antibodies. The band intensity was quantified and converted to ng TK2 protein per mg mitochondrial protein using recombinant human TK2 as standard. The results are presented as mean ± SD. A representative image from IP-western blot is shown. COX 4 was used as loading control in crude extracts before immunoprecipitation. Numbers 1 to 7 represents liver, heart, brain, kidney, spleen, lung and skeletal muscle

Mitochondrial and cytosolic thymidine kinase activities were determined using tritium labelled dT as substrate [29]. High levels of mitochondrial TK2 activity was detected in brain, spleen and lung mitochondria, while intermediate levels were found in liver, heart and kidney mitochondria; in skeletal muscle mitochondria TK2 activity was the lowest. In most tissues the cytosolic dT kinase activity was very low as compared with the mitochondrial TK2 activity, except in spleen which had the highest cytosolic dT kinase activity due to the presence of TK1. Overall spleen had the highest and skeletal muscle had the lowest total thymidine kinase activity (Fig. 4a).

### The concentrations of mitochondrial TK2 protein

Mitochondrial TK2 protein was determined by an immunoprecipitation method using anti-TK2 antibodies. Recombinant human mitochondrial TK2 was used as standard in subsequent western blot in order to quantify the level of mitochondrial TK2. As shown in Fig. 4b mitochondrial TK2 protein levels were in the range of 20–60 ng per mg total mitochondrial protein. Liver mitochondria contained the highest amount of TK2 protein, heart and spleen mitochondria contained ~ 2/3 of that in liver mitochondria. In brain, kidney, lung and skeletal muscle the TK2 levels were similar, and was ~ 1/3 of that of liver. These results demonstrated a lack of correlation between TK2 activity and protein levels and suggested that a post-translational regulation mechanism may be in operation.

### Ribonucleotide reductase (RNR) and p53R2

**Fig. 5:**
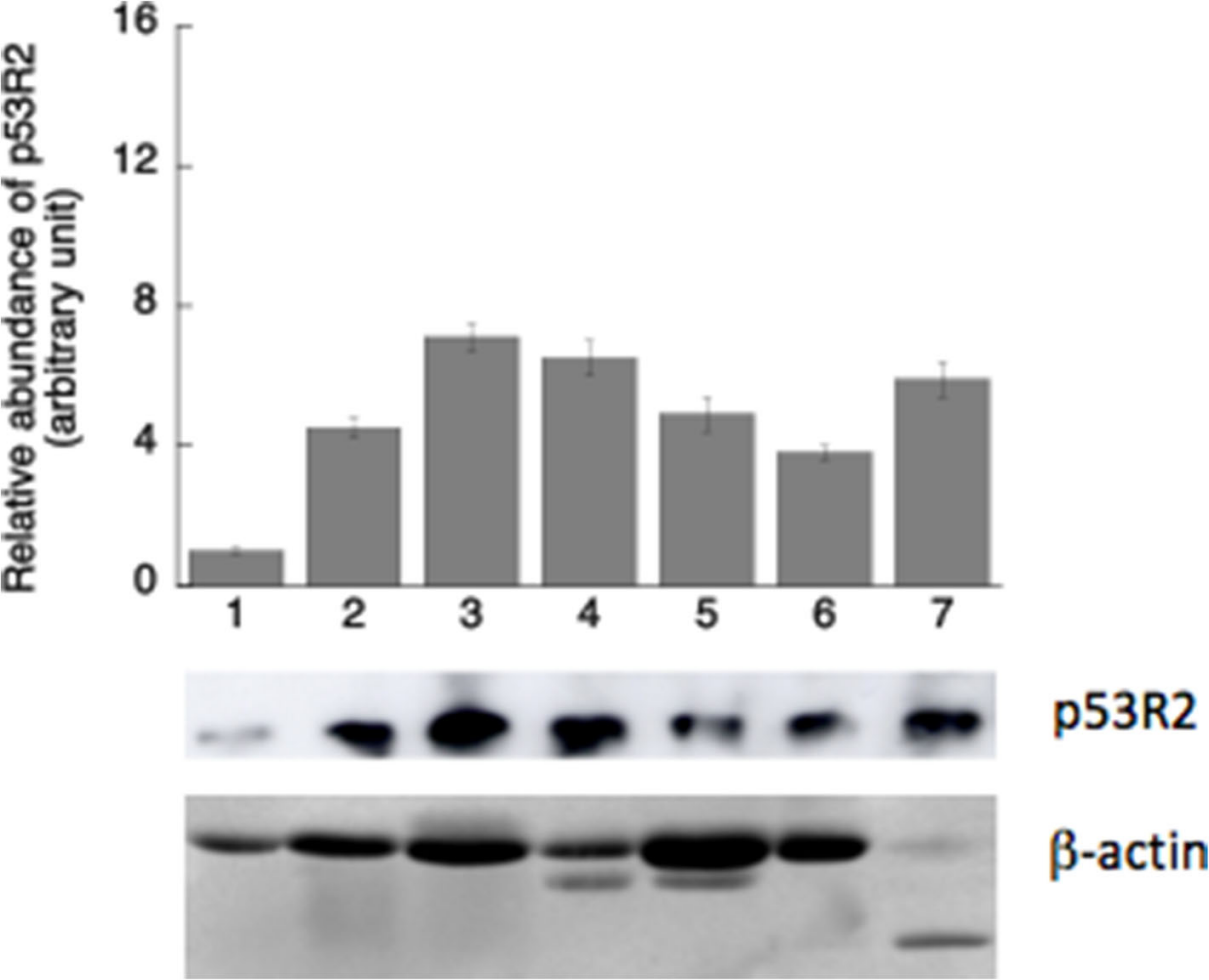
p53R2 protein level in cytosolic extracts determined by immunoprecipitation and western blot analysis. The band intensity was quantified and shown as mean ± SD. A representative image from IP-western blot is shown. β-actin was used as loading control in crude extracts before immunoprecipitation. Numbers 1 to 7 represents liver, heart, brain, kidney, spleen, lung and skeletal muscle

An attempt to measure RNR activity using radiolabelled CDP was not successful, probably due to low levels of RNR activity and/or interfering enzymes in crude extracts. In order to estimate the cytosolic RNR activity we determined the p53R2 protein concentrations in cytosolic and mitochondrial extracts by using immunoprecipitation method with p53R2 specific antibodies. In the cytosolic extracts the p53R2 levels were similar in brain, kidney, skeletal muscle, heart, spleen and lung, but lower in liver (Fig. 5). No p53R2 protein could be detected in the mitochondrial extracts.

### Thymidylate synthase

**Fig. 6:**
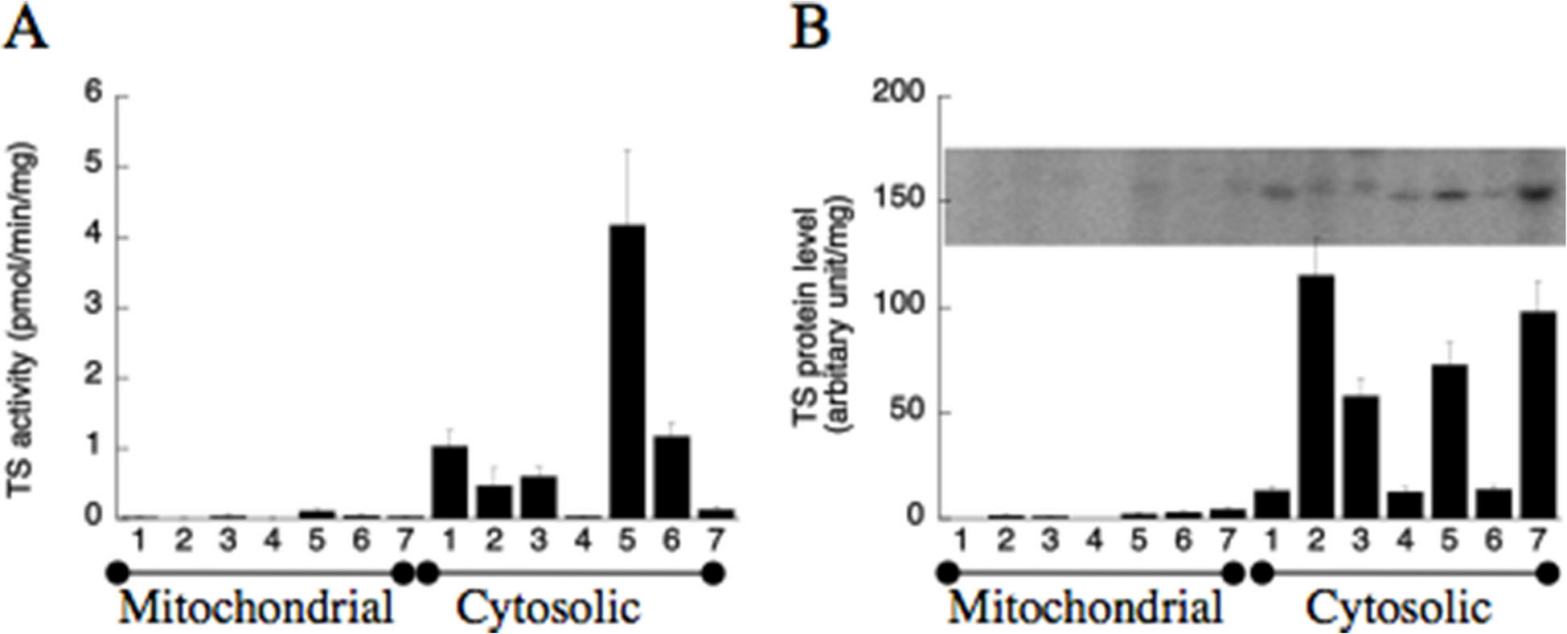
Thymidylate synthase activity (**a**) and protein level (**b**) in rat tissues. Equal volume of cytosolic and mitochondrial extracts was used in the TS activity (expressed as pmol/min/mg) and radiolabelling of TS protein experiments using [^32^P]-5-fluoro-dUMP. The intensity of each band was quantified and normalized to the amount of protein used in the experiments and expressed as arbitrary unit/mg protein. A representative autoradiograph image is shown. Results are mean ± SD. Number 1 to 7 represents liver, heart, brain, kidney, spleen, lung and skeletal muscle

The cytosolic TS activity was highest in spleen and lowest in kidney. Intermediate TS activity was found in liver, lung, heart and brain, and low activity in skeletal muscle and kidney (Fig. 6a). There was no detectable TS activity in mitochondrial extracts from most tissues, except that a low TS activity was detected in spleen mitochondrial extracts, which might be due to contamination by cytosolic TS (Fig. 6a).

TS protein level was high in heart, brain, spleen and skeletal muscle but low in liver, kidney, and lung extracts (Fig. 6b). There was no detectable TS protein in mitochondrial extracts from liver, heart, brain, and kidney but a very low level of TS protein was detected in spleen and skeletal muscle (Fig. 6b). Furthermore, TS protein detected in heart and brain showed higher molecular mass on SDS-PAGE as compared with other tissues (Fig. 6b), suggesting post-translational modifications. In heart and skeletal muscle TS protein levels were high but activity levels were low. The reason for this discrepancy is not known but other factors such as post-translation modifications may affect TS activity. As shown in earlier studies, phosphorylation on Ser-12 [30] or S-glutathionylation on Cys-195 lead to decreased activity [31].

## Discussion

Defect nucleotide metabolism causes devastating MDS and also nuclear genome instability [19–21, 32]. Current deoxynucleoside based therapy has been shown to improve the quality of life and lifespan in human patients and animal models [33–35]. To understand how this treatment works we need to study the enzymes in nucleotide metabolism.

Here we have determined the levels of TKs, TS and p53R2 in adult rat tissues at the sub-cellular level in order to estimate the capacity of the salvage and the de novo synthesis of dTMP in normal tissues. We have also clarified which cytosolic enzyme e.g. TK1, TK2, and dCK is present in different tissues, and characterized a putative cytosolic TK2-like enzyme partially purified from brain and heart. The cytosolic TK2-like enzyme showed similar substrate specificity as the mitochondrial form. At present we do not know if the cytosolic TK2-like enzyme is an alternative spliced form of mitochondrial TK2 or encoded by a new gene, since in most human tissues multiple mitochondrial TK2 mRNA transcripts exist [27, 36] and a TK2 isoform lacking mitochondrial targeting sequence has been reported previously [36] in addition to the full length TK2 sequence with mitochondrial leader sequence [5, 28]. Nonetheless we show here that the kinetic properties of the two variant TK2s are very similar, and further investigation of the cytosolic TK2-like enzyme is warranted. If the cytosolic TK2-like enzyme described here is encoded by a different gene it may contribute to and help to explain the therapeutic effects of deoxynucleoside treatment in TK2 knock-out mouse models as well as in human patients with TK2 deficiency [33–35, 37, 38]. Furthermore, the presence of cytosolic dCK may also contribute to the salvage synthesis of thymidine nucleotide (Fig. 1).

Mitochondrial TK2 activity was dominating in all tissues as compared with the cytosolic TK2-like activity [7]. Although there is a large variation in the mitochondrial TK2 activity from tissue to tissue, mitochondrial TK2 protein levels showed only 2 to 3-fold difference. The lack of correlation between mitochondrial TK2 activity and protein concentrations in liver, heart and skeletal muscle is most likely due to the high respiration rates in these tissues. This may result in oxidative stress induced modifications that inactivate and/or degrade the mitochondrial TK2 protein as demonstrated earlier [39]. Nonetheless the specific activity of mitochondrial TK2 determined here in rat tissues showed a clear correlation to the levels of dTTP pools but not dCTP pools determined previously in rat liver, heart, brain and skeletal muscle mitochondria [32, 40].

The de novo synthesis of dNTPs relies on RNR activity and in non-proliferating tissues the RNR activity is dependent on the expression of p53R2 [10, 12]. In all tissues p53R2 levels were similar except liver for which the p53R2 level was low. For the de novo synthesis of dTTP TS activity is required. High TS activity was found in spleen, but low in all other tissues, indicating a generally low capacity of de novo dTMP synthesis.

Using tissue mRNA expression data from Expression Atlas [41] for Sprague Dawley we compared the mRNA levels of TK2, TS and p53R2 with protein concentrations determined here in the corresponding tissue and could not find clear-cut correlations (Fig. S3. A, B, C). The lack of correlation between mRNA and protein could also be seen in case of TK1 and dCK; high level of TK1 mRNA was found in kidney, spleen, lung and liver, however, TK1 protein was mainly detected in spleen and liver but not kidney and lung; dCK mRNA was highly expressed in brain but we could not detected any dCK protein (Fig. S3. D). The lack of correlation between the levels of mRNA and protein may be explained by post-translational modifications such as S-glutathionylation affecting protein stability [30, 31, 39].

In mice, p53R2 knockout results in mtDNA depletion, however, over expression of p53R2 alone or in combination with Rrm1 (coding for ribonucleotide reductase R1 subunit) lead to increased dATP and dCTP pools but surprisingly a decreased dTTP pool in skeletal muscle [16, 42]. In TK2 knockout mice a significant decrease in dTTP but not dCTP pools was observed in heart and brain tissues [13]. All these results point to that de novo synthesis can provide sufficient dCTP but not dTTP for mtDNA synthesis. Furthermore, in most non-proliferating tissues TK2 is apparently responsible for the overall dTMP synthesis needed for mtDNA replication and nuclear DNA repair [43].

## Conclusions

This study suggests that the mitochondrial TK2 and the cytosolic TK2-like enzymes are the main enzymes for the synthesis of pyrimidine nucleotides in non-proliferating tissues; while TK1 and dCK contribute to pyrimidine nucleotides synthesis in tissues with high fractions of proliferating cells. The limiting factor in the de novo synthesis of dTMP is the level of TS activity. Of course different species may have differences in the expression and levels of these enzymes and the observed phenotypic differences between TK2 knock-out mice and TK2-deficient human patients strongly indicate tissue specificity differences between the two species. Recent studies in TK2 H126N knock-in and TK2 knock-out mice clearly substantiate this conclusion [38, 44]. Future studies of these human enzymes will most likely aid improvements of the therapeutic approaches for mitochondrial disorders caused by defect in nucleotide metabolism.

## Methods

### Materials

Radioactive substances [methyl-^3^H]-thymidine (dT, 20 Ci/mmol) and [γ-^32^P] ATP (3000 Ci/mmol) were obtained from Perkin Elmer. [5-^3^H]-deoxycytidine (dC, 27 mCi/ml) and [5-^3^H]-deoxyuridine monophosphate (dUMP, 24 Ci/mmol) were from Moravek Biochemicals, Inc. [^32^P]-5FdUMP (5-fluorodeoxyuridine 5′-monophosphate) was prepared from 5FdUrd (5-fluorodeoxyuridine) and [γ-^32^P]-ATP in an enzymatic reaction catalysed by thymidine kinase. Antibodies against cytochrome c oxidase subunit 4 (COX 4) and cytochrome c were purchased from CLONTECH. Goat anti-TK2 antibodies, rabbit anti-R2 and goat anti-p53R2 antibodies were from Santa Cruz biotechnology, Inc. DTNB (5,5′-dithiobis(2-nitrobenzoic acid)) and Oxaloacetate were from Sigma-Adrich. Acetyl-CoA was from Roche Diagnostics GmbH.

### Enzyme assays

Deoxynucleoside kinase activity was determined by using either [^3^H]-dT or [^3^H]-dC as substrate as described previously [27] with the addition of 6-chloro-5 amino-uracil (1 μM) and NaF (15 mM) to inhibit thymidine phosphorylase and 5′-nucleotidase activities. Thymidylate synthase activity was determined by using the tritium release assay [45] with [5-^3^H]-dUMP as substrate. NaF (15 mM) was added to inhibit 5′-nucleotidase/phosphatase activity. All the assays were performed in triplicates for each tissue type from individual animal and the results were presented as mean ± SD in pmol product formed/min/mg.

Citrate synthase activity was determined in both mitochondrial and cytosolic extracts at room temperature (21 °C) as previously described [46]. Briefly, mitochondrial and cytosolic extracts (40 μg) were added to a reaction mixture containing 0.2 M Tris/HCl, pH 8.0, 0.1 mM DTNB (5,5′-dithiobis[2-nitrobenzoic acid], and 0.3 mM Acetyl-CoA in a total volume of 475 μl and incubated for 2 min, and then the reaction was started by addition of 25 μl Oxaloacetate (10 mM). The formation of product was monitored at 412 nm over 3 min in a spectrophotometer. The assays were repeated four times and the results were presented as mean ± SD.

### Isolation of intact mitochondria and cytosol

Mitochondria and cytosol were prepared by differential centrifugation method [22] using fresh tissues from adult rats (Sprague Dawley, 10 weeks old, purchased from Scanbur LabAnimal, Danmark, *n* = 5). The rats were housed at animal facility at Uppsala University according to international regulations and the experiment was approved by the local ethical committee in Uppsala, Sweden. The rats were sacrificed by using a mixture of medicine air and successive increase of carbon dioxide in the cage. Organs were removed and used directly. The cytosol and mitochondria preparations were stored in aliquots at − 70 °C until further analysis. Mitochondrial proteins were extracted by sonication in ice/water bath after addition of 0.5% NP-40 (Nonidet P-40). After centrifugation (16,000 x g for 20 min at 4 °C) the clear supernatant was used in the assays. Protein concentrations were determined by the Bio-Rad protein assay using bovine serum albumin as standard.

### Anionic and cationic exchange chromatography and partial purification of a cytosolic form of a TK2-like enzyme

Cytosolic extracts were applied to a pre-equilibrated anionic exchange column (DEAE fast flow, GE Healthcare, column size 1 ml) in buffer A (25 mM Tris/HCl, pH 7.6, 2 mM MgCl_2_, 1 mM Dithiothreitol (DTT) and 15% Glycerol) and eluted with linear gradient (0–0.3 M) of KCl in buffer A. Fractions (0.5 ml) were collected and assayed for dT and dC kinase activity. In case of the cationic exchange chromatography the flow through from DEAE-Sephadex column was collected and applied to a pre-equilibrated CM-Sepharose column (GE Healthcare, column size 1 ml) in buffer A and eluted with linear gradient (0–0.3 M) of KCl in buffer A. Fractions (0.5 ml) were collected and assayed for dT and dC kinase activity.

Fractions containing TK2 activity from the DEAE chromatography of brain and heart cytosolic extracts were pooled, the protein concentration was determined and used in kinetic studies with tritium labelled dT and dC as the variable substrate at fixed ATP concentration. The kinetic parameters were calculated by using the SigmaPlot enzyme kinetics module 1.1 (SPSS Science).

### Western blots analysis and immunoprecipitation

Mitochondrial and cytosolic proteins (10 μg/lane) were separated on 14% SDS-polyacrylamide gels and transferred to polyvinylidene difluoride (PVDF) membranes, which were then probed for 2 h with anti-cytochrome c oxidase subunit 4 (COX 4) (1:500 dilution) and anti-cytochrome c (1:200 dilution), or anti-β-actin (1:1000 dilution) antibodies at room temperature and detected by the enhanced chemiluminescence detection system (GE healthcare).

Immunoprecipitation was performed with 1 mg cytosolic or mitochondrial proteins following standard protocols. A rabbit anti-R2 antibody or goat anti TK2 antibody was added to the cytosolic respective mitochondrial extracts and incubated over night at 4 °C. Protein A Sepharose was added and incubated for additional 2 h, then collected by centrifugation. After washing 3 times with PBS, 50 μl SDS sample buffer was added to the slurry. After boiling for 5 min and brief centrifugation 20 μl supernatant was applied to 12% SDS- polyacrylamide gels. After electrophoresis the proteins were transferred to PVDF membranes. A goat anti-p53R2 antibody or rabbit anti-TK2 antibody was used in western blot to detect p53R2 protein or TK2 protein, respectively. Band intensities were quantified by using Image Gauge software version 3.3 (Fuji Photofilm Co. Ltd). In order to quantify mitochondrial TK2 protein in the western,blots recombinant human TK2 (2, 5, 10, and 25 ng) were loaded on the same gel and the band intensity was quantified and a standard curve was constructed (supplementary figure S2). The amount of TK2 in these tissue samples was calculated based on the standard curve. The results were presented as mean ± SD in arbitrary units/mg protein in case of p53R2, and ng/mg protein in case of mitochondrial TK2 protein using recombinant TK2 as standard.

### Determination of TS protein levels

5-fluoro-2′-deoxyuridine-5′-monophosphate (5FdUMP) forms a covalent ternary complex with the TS protein in the presence of methylene tetrahydrofolate. Therefore, we used [^32^P]-5FdUMP to determine the levels of TS. Mitochondrial and cytosolic extracts (20 μl) were mixed with 10 μl reaction mixture containing 25 mM MgCl_2_, 0.2 mM methylene tetrahydrofolate, 0.2 mM Cytidine 5′-monophosphate, 20 mM DTT and 5 μM [^32^P]-5FdUMP. The mixtures were incubated at 37 °C for 2 h and then stopped by addition of the SDS sample buffer (5 μl). After heating at 95 °C for 3 min 15 μl was loaded onto 12% SDS-PAGE gels. After electrophoresis the gels were fixed in methanol/acetic acid and the radiolabelled protein bands were visualized by autoradiography. The band intensity was quantified by using Image Gauge software version 3.3 (Fuji Photofilm Co. Ltd). The results were presented as mean ± SD in arbitrary unit/mg protein.

## Supplementary information

**Additional file 1: Table S1**. dT and dC kinase activity in mitochondrial and cytosolic extracts. **Figure S1.** Assessment of the quality of mitochondrial and cytosolic preparations by using western blot analyses using anti cytochrome c oxidase subunit 4 (COX4) and anti-cytochrome c antibodies. Mitochondrial and cytosolic preparation from liver, heart, brain, spleen, kidney, and skeletal muscle (A); and lung and heart (B). **Figure S2**. Recombinant TK2 was used as standard in western blot analysis for determination of mitochondrial TK2 in rat tissue mitochondrial extracts. TK2 (2, 5, 10, and 25 ng) was loaded onto the same gel for western blot analysis using TK2 specific antibody and the band intensity was quantified and plotted against the amount of TK2 protein in each lane to generate a standard curve by using linear regression analysis. **Figure S3.** Direct correlation analysis of mRNA levels of TK2 (A), p53R2 (B) and TS (C) with protein concentration determined by western blot analyses (TK2 and p53R2) and radiolabeling (TS). Tissues mRNA expression data/heat map is from Expression Atlas (D)

## Data Availability

All data generated in this study are included in the manuscript and supplementary material.

## Abbreviations

TK2: Mitochondrial thymidine kinase 2
TK1: Cytosolic thymidine kinase 1
TS: Thymidylate synthase
dCK: Deoxycytidine kinase
p53R2: P53 inducible ribonucleotide reductase small subunit
dTMP: Thymidylate or thymidine 5′-monophosphate
dUMP: 2′-deoxyuridine 5′-monophosphate
dNTP: Deoxynucleoside triphosphate
5FdUMP: 5-fluoro-2′-deoxyuridine 5′-monophosphate
dT: Thymidine
dC: 2′-deoxycytidine
COX4: Cytochrome oxidase subunit 4
mtDNA: Mitochondrial DNA
MDS: MtDNA depletion syndrome

## References

[1] Gasparri F, Wang N, Skog S, Galvani A, Eriksson S (2009). Thymidine kinase 1 expression defines an activated G1 state of the cell cycle as revealed with site-specific antibodies and ArrayScan assays. Eur J Cell Biol.

[2] Ke PY, Chang ZF (2004). Mitotic degradation of human thymidine kinase 1 is dependent on the anaphase-promoting complex/cyclosome-CDH1-mediated pathway. Mol Cell Biol.

[3] Eriksson S, Wang L (2008). Molecular mechanisms of mitochondrial DNA depletion diseases caused by deficiencies in enzymes in purine and pyrimidine metabolism. Nucleosides Nucleotides Nucleic Acids.

[4] Sun R, Eriksson S, Wang L (2014). Mitochondrial thymidine kinase 2 but not deoxyguanosine kinase is up-regulated during stationary growth phase of the cultured cells. Nucleosides Nucleotides Nucleic Acids.

[5] Wang L, Eriksson S (2000). Cloning and characterization of full length mouse thymidine kinase 2: the N-terminal sequence directs import of the precursor protein into mitochondria. Biochem J.

[6] Mirzaee S, Eriksson S, Albertioni F (2010). Differences in cytosolic and mitochondrial 5′-nucleotidase and deoxynucleoside kinase activities in Sprague-Dawley rat and CD-mouse tissues. Toxicology.

[7] Wang L, Eriksson S (2010). Tissue specific distribution of pyrimidine deoxynucleoside salvage enzymes shed light on the mechanism of mitochondrial DNA depletion. Nucleosides Nucleotides Nucleic Acids.

[8] Spasokoukotskaja T, Arnér E, Brosjö O, Gunven P, Juliusson G, Liliemark J, Eriksson S (1995). Expression of deoxycytidine kinase and phosphorylation of 2-chlorodeoxyadenosine in human normal and tumour cells and tissues. Eur J Cancer.

[9] Arnér ESJ, Eriksson S (1995). Mammalian deoxyribonucleoside kinases. Pharmacol Ther.

[10] Eriksson S, Gräslund A, Skog S, Thelander L, Tribukait B (1984). Cell cycle-dependent regulation of mammalian ribonucleotide reductase. The S phase-correlated increase in subunit M2 is regulated by de novo protein synthesis. J Biol Chem.

[11] Engström Y, Eriksson S, Jildevik I, Skog S, Thelander L, Tribukait B (1985). Cell cycle-dependent expression of mammalian ribonucleotide reductase: differential regulation of the two subunits. J Biol Chem.

[12] Håkansson P, Hofer A, Thelander L (2006). Regulation of mammalian ribonucleotide reduction and dNTP pools after DNA damage and in resting cells. J Biol Chem.

[13] Dorado B, Area E, Akman H, Hirano M (2011). Onset and organ specificity of TK2 deficiency depends on TK1 down-regulation and transcriptional compensation. Hum Mol Genet.

[14] Herzfeld A, Raper SM (1980). Relative activities of thymidylate synthase and thymidine kinase in rat tissues. Cancer Res.

[15] Saada A, Shaag A, Mandel H, Nevo Y, Eriksson S, Elpeleg O (2001). Mutant mitochondrial thymidine kinase in mitochondrial DNA depletion myopathy. Nat Genet.

[16] Bourdon A, Minai L, Serre V, Jais J-P, Sarzi E, Aubert S, Chretien D, de Lonlay P, Paquis-Flucklinger V, Arakawa H, Nakamura Y, Munnich A, Rötig A (2007). Mutation of RRM2B, encoding p53-controlled ribonucleotide reductase (p53R2), causes severe mitochondrial DNA depletion. Nat Genet.

[17] Akman H, Dorado B, López L, García-Cazorla A, Vilà M, Tanabe L, Dauer W, Bonilla E, Tanji K, Hirano M (2008). Thymidine kinase 2 (H126N) knockin mice show the essential role of balanced deoxynucleotide pools for mitochondrial DNA maintenance. Hum Mol Genet.

[18] Zhou X, Solaroli N, Bjerke M, Stewart J, Rozell B, Johansson M, Karlsson A (2008). Progressive loss of motochondrial DNA in thymidine kinase 2 deficient mice. Hum Mol Genet.

[19] Niida H, Shimada M, Murakami H, Nakanishi M (2010). Mechanisms of dNTP supply that play an essential role in maintaining genome integrity in eukaryotic cells. Cancer Sci.

[20] Pai C, Kearsey S (2017). A critical balance: dNTPs and the maintenance of genome stability. Genes.

[21] Hämäläinen RH, Landoni JC, Ahlqvist KJ, Goffart S, Ryytty S, Obaidur Rahman M, Brilhante V, Icay K, Hautaniemi S, Wang L, Laiho M, Suomalainen A (2019). Defects in mtDNA replication challenge nuclear genome stability through nucleotide depletion and provide a unifying mechanism for mouse progerias. Nat Metab.

[22] Graham JM. Subcellular fractionation and isolation of organelles. In: Current Protocol in Cell Biology. Wiley; 1999. p. 3.3.1–3.3.15.

[23] Palmer JW, Tandler B, Hoppel CL (1977). Biochemical properties of subsarcolemmal and interfibrillar mitochondria isolated from rat cardiac muscle. J Biol Chem.

[24] Wang L, Karlsson A, Arnér ESJ, Eriksson S (1993). Substrate specificity of mitochondrial 2′-deoxyguanosine kinase efficient phosphorylation of 2-chlorodeoxyadenosine. J Biol Chem.

[25] Bohman C, Eriksson S (1988). Deoxycytidine kinase from human leukemic spleen: preparation and characterization of the homogeneous enzyme. Biochemistry.

[26] Munch-Petersen B, Cloos L, Tyrsted G, Eriksson S (1991). Diverging substrate specificity of pure human thymidine kinases 1 and 2 against antiviral dideoxynucleosides. J Biol Chem.

[27] Wang L, Munch-Petersen B, Herrström Sjöberg A, Hellman U, Bergman T, Jörnvall H, Eriksson S (1999). Human thymidine kinase 2: molecular cloning and characterisation of the enzyme activity with antiviral and cytostatic nucleoside substrates. FEBS Lett.

[28] Wang L, Saada A, Eriksson S (2003). Kinetic properties of mutant human thymidine kinase 2 suggest a mechanism for mitochondrial DNA depletion myopathy. J Biol Chem.

[29] Wang L, Eriksson S (2008). 5-Bromovinyl 2′-deoxyuridine phosphorylation by mitochondrial and cytosolic thymidine kinase (TK2 and TK1) and its use in selective measurement of TK2 activity in crude extracts. Nucleosides Nucleotides Nucleic Acids.

[30] Jarmula A, Fraczyk T, Cieplak P, TRode W (2010). Mechanism of influence of phosphorylation on serine 124 on a decrease of catalytic activity of human thymidylate synthase. Bioorg Med Chem.

[31] Gibson LM, Celeste LR, Lovelace LL, Lebioda L (2011). Structures of human thymidylate synthase R163K with dUMP, FdUMP and glutathione show asymmetric ligand binding. Acta Crystallogr D Biol Crystallogr.

[32] Wang L (2010). Deoxynucleoside salvage enzymes and tissue specific mitochondrial DNA depletion. Nucleosides Nucleotides Nucleic Acids.

[33] Marti R (2016). Nucleosides to treat mitochondrial DNA maintenance defects. Neuromuscl Disord.

[34] Lopez-Gomez C, Levy R, Sanchez-Quintero M, Juanola-Falgarona M, Barca E, Garcia-Diaz B, Tadesse S, Garone C, Hirano M (2017). Deoxycytidine and deoxythymidine treatment for thymidine kinase 2 deficiency. Ann Neurol.

[35] Dominguez-Gonzalez C, Madruga-Garrido M, Mavillard F, Garone C, Aguirre-Rodriguez F, Donati M, Kleinsteuber K, Marti I, Marti-Hernandez E, Morealejo-Aycinena J, Munell F, Nascimento A, Kalko S, Sardina M, Alvarez del Vayo C, Serrano O, Long Y, Tu Y, Levin B, Thompson J, Engelstad K, Uddin J, Torres-Torronteras J, Jimenez-Mallebrera C, Marti R, Paradas C, Hirano M (2019). Deoxynucleotide therapy for thymidine kinase 2 -deficient myopathy. Ann Neurol.

[36] Johansson M, Karlsson A (1997). Cloning of the cDNA and chromosome localization of the gene for human thymidine kinase 2. J Biol Chem.

[37] Camara Y, Gonzalez-Vioque E, Scarpelli M, Torres-Torronteras J, Caballero A, Hirano M, Martı R (2014). Administration of deoxyribonucleosides or inhibition of their catabolism as a pharmacological approach for mitochondrial DNA depletion syndrome. Hum Mol Genet.

[38] Blazquez-Bermejo C, Molina-Granada D, Vila-Julia F, Jimenez-Heis D, Zhao X, Torres-Torronteras J, Karlsson A, Marti R, Camara Y (2019). Age-related metabolic changes limit efficacy of deoxynucleoside-based therapy in thymidine kinase 2-deficient mice. EbioMedicine.

[39] Sun R, Eriksson S, Wang L (2012). Oxidative stress induced S-glutathionylation and proteolytic degradation of mitochondrial thymidine kinase 2. J Biol Chem.

[40] Song S, Pursell Z, Copeland W, Longley M, Kunkel T, Mathews C (2005). DNA precursor asymmetries in mammalian tissue mitochondria and possible contribution to mutagenesis through reduced replication fidelity. Proc Natl Acad Sci U S A.

[41] Expression Atlas: https://www.ebi.ac.uk/gxa/experiments/E-MTAB-2800/Results?geneQuery=%5B%7B%22value%22%3A%22ensrnog00000012853%22%7D%5D&filterFactors=%7B%22STRAIN%22%3A%5B%22Sprague-Dawley%22%5D%7D.

[42] Ylikallio E, Page JL, Xu X, Lampinen M, Bepler G, Ide T, Tyynismaa H, Weiss RS, Suomalainen A (2010). Ribonucleotide reductase is not limiting for mitochondrial DNA copy number in mice. Nucleic Acids Res.

[43] Lee M-H, Wang L, Chang Z-F (2014). The contribution of mitochondrial thymidylate synthesis in preventing the nuclear genome stress. Nucleic Acids Res.

[44] Lopez-Gomez C, Hewan H, Sirra C, Akman H, Sanchez-Quintero M, Juanola-Falgarona M, Tadesse S, Tanji K, Konofagou E, Hirano M (2019). Bioavailability and cytosolic kinases modulate response to deoxynucleoside therapy in TK2 deficiency. EbioMedicine.

[45] Armstrong RD, Diasio RB (1982). Improved measurement of thymidylate synthetase activity by a modified tritium-release assay. J Biochem Biophys Methods.

[46] Spinnazzi M, Casarin A, Pertegato V, Salviati L, Angelini C (2012). Assessment of mitochondrial respiratory chain enzymatic activities on tissues and cultured cells. Nat Protocols.

