# Supplementary material for: Basic biochemical characterization of cytosolic enzymes in thymidine nucleotide synthesis in adult rat tissues: implications for tissue specific mitochondrial DNA depletion and deoxynucleoside-based therapy for TK2-deficiency
Source: BMC Mol Cell Biol. 2020 Apr 28;21:33. doi: 10.1186/s12860-020-00272-3 (PMC7189545; doi:10.1186/s12860-020-00272-3)
Supplement: Supplementary file 1 — Additional file 1: Table S1. dT and dC kinase activity in mitochondrial and cytosolic extracts. Figure S1. Assessment of the quality of mitochondrial and cytosolic preparations by using western blot analyses using anti cytochrome c oxidase subunit 4 (COX4) and anti-cytochrome c antibodies. Mitochondrial and cytosolic preparation from liver, heart, brain, spleen, kidney, and skeletal muscle (A); and lung and heart (B). Figure S2. Recombinant TK2 was used as standard in western blot analysis for determination of mitochondrial TK2 in rat tissue mitochondrial extracts. TK2 (2, 5, 10, and 25 ng) was loaded onto the same gel for western blot analysis using TK2 specific antibody and the band intensity was quantified and plotted against the amount of TK2 protein in each lane to generate a standard curve by using linear regression analysis. Figure S3. Direct correlation analysis of mRNA levels of TK2 (A), p53R2 (B) and TS (C) with protein concentration determined by western blot analyses (TK2 and p53R2) and radiolabeling (TS). Tissues mRNA expression data/heat map is from Expression Atlas (D) [file 12860_2020_272_MOESM1_ESM.docx]

Supplementary material

Basic biochemical characterization of cytosolic thymidine nucleotide biosynthesis enzymes in adult rat tissues: implications for tissue specific mitochondrial DNA depletion and deoxynucleoside-based therapy for TK2-deficiency

**Liya Wang*, Ren Sun and Staffan Eriksson**

Department of Anatomy, Physiology and Biochemistry, Swedish University of Agricultural Sciences, SE-750 07 Uppsala, Sweden

*Corresponding author: Dr. Liya Wang, Tel: +46 18 672820; and e-mail: [liya.wang@slu.se](mailto:liya.wang@slu.se)

Table S1 dT and dC kinase activity in mitochondrial and cytosolic extracts.

| Tissue | Mitochondrial | | Cytosolic | |
| --- | --- | --- | --- | --- |
|  | dT | dC | dT | dC |
| Liver | 2.1 ± 1.0 (2.6) | 2.8 ± 1.1 | 0.16 ± 0.08 (0.9) | 0.74 ± 0.1 |
| Heart | 3.1 ± 0.8 (3.9) | 2.5 ± 0.9 | 0.41 ± 0.03 (2.4) | 0.46 ± 0.1 |
| Brain | 9.1 ± 1.3 (11.3) | 9.7 ± 1.4 | 0.83 ± 0.4 (4.9) | 1.6 ± 0.8 |
| Kidney | 2.3 ± 0.7 (2.9) | 3.6 ± 1.1 | 0.22 ± 0.06 (1.3) | 0.72 ± 0.1 |
| Spleen | 11.2 ± 2.8 (14) | 13.1 ± 5.2 | 13.6 ± 3.6 (80) | 6.7 ± 0.5 |
| Lung | 6.3 ± 0.2 (7.9) | 9.8 ± 0.3 | 0.4 ± 0.09 (2.4) | 2.6 ± 0.9 |
| Skeletal muscle | 0.80 ± 0.2 (1.0) | 0.95 ± 0.2 | 0.17 ± 0.1 (1.0) | 0.14 ± 0.08 |

Assays were performed with [^3^H]-dT and [^3^H]-dC as substrate and mitochondrial and cytosolic extracts as enzyme source. The samples were run in duplicates for mitochondrial and cytosolic preparation from each animal. Total number of rats was 5. The activity is given as (pmol/min/mg) mean ± SD.

Data in parenthesis indicates fold differences of dT kinase activity as compared with that of the skeletal muscle (set to 1.0).

Figure S1


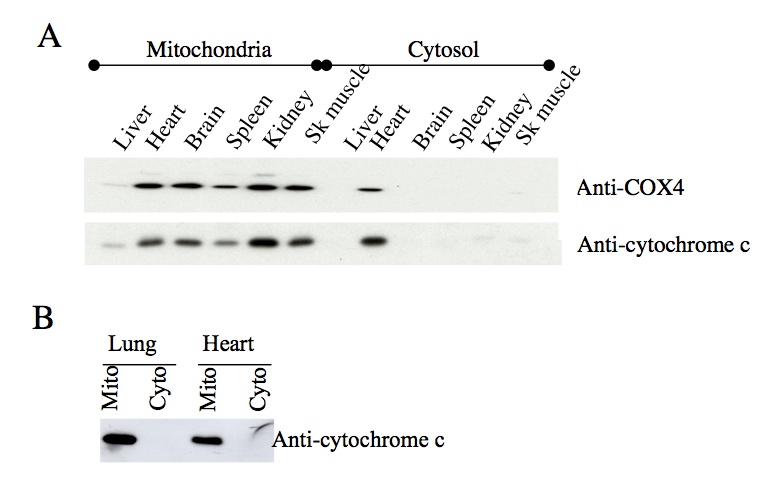


Figure S1. Assessment of the quality of mitochondrial and cytosolic preparations by using western blot analyses using anti cytochrome c oxidase subunit 4 (COX4) and anti-cytochrome c antibodies. Mitochondrial and cytosolic preparation from liver, heart, brain, spleen, kidney, and skeletal muscle (A); and lung and heart (B).

Figure S2


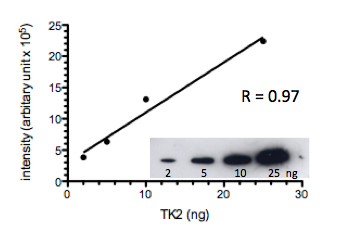


Figure S2. Recombinant TK2 was used as standard in western blot analysis for determination of mitochondrial TK2 in rat tissue mitochondrial extracts. TK2 (2, 5, 10, and 25 ng) was loaded onto the same gel for western blot analysis using TK2 specific antibody and the band intensity was quantified and plotted against the amount of TK2 protein in each lane to generate a standard curve by using linear regression analysis.

Figure S3


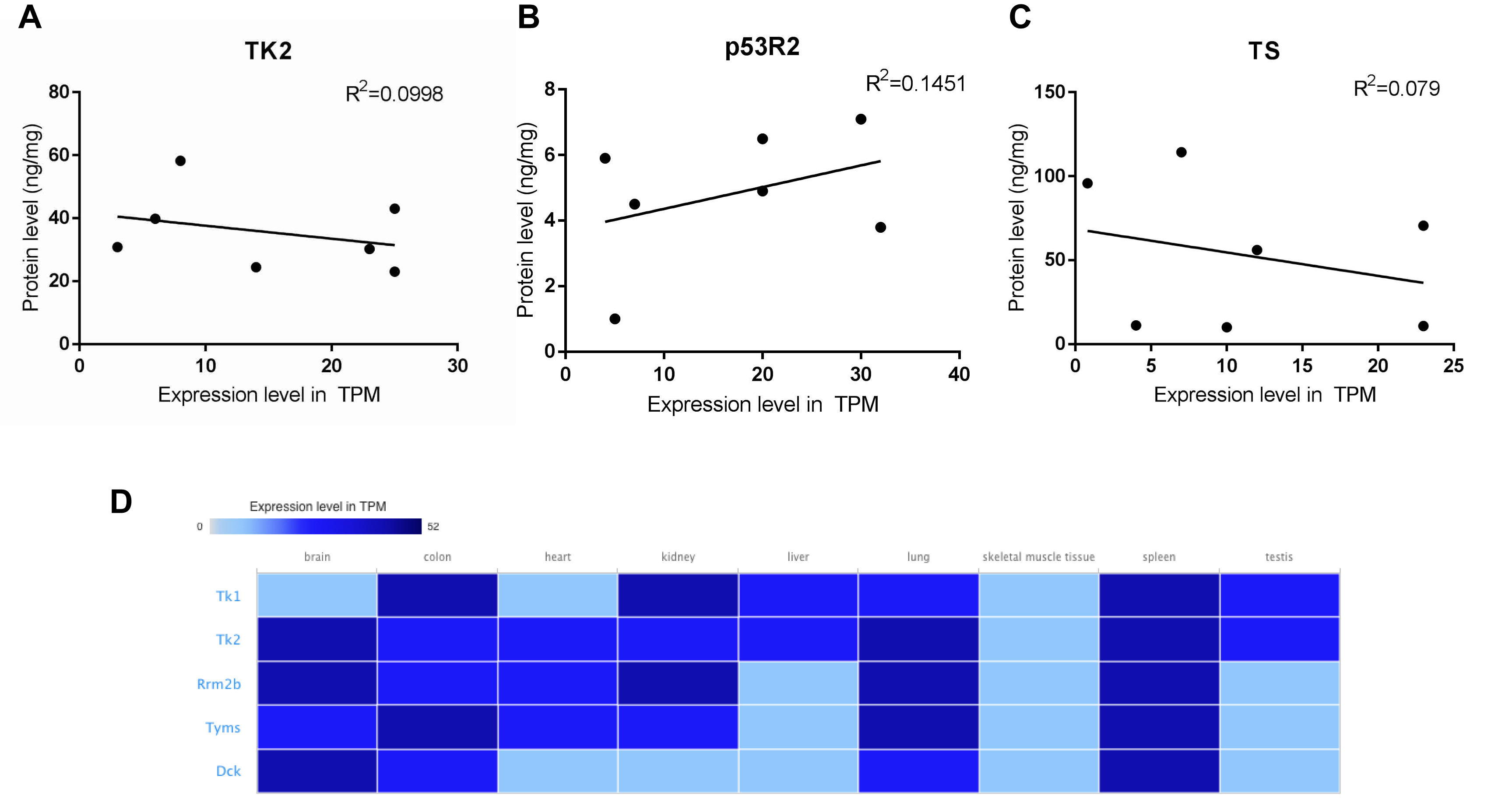


Figure S3. Direct correlation analysis of mRNA levels of TK2 (A), p53R2 (B) and TS (C) with protein concentration determined by western blot analyses (TK2 and p53R2) and radiolabeling (TS). Tissues mRNA expression data/heat map is from Expression Atlas (D).
